# Super-resolution deep learning image reconstruction: image quality and myocardial homogeneity in coronary computed tomography angiography

**DOI:** 10.1186/s44348-024-00031-4

**Published:** 2024-09-20

**Authors:** Chuluunbaatar Otgonbaatar, Hyunjung Kim, Pil-Hyun Jeon, Sang-Hyun Jeon, Sung-Jin Cha, Jae-Kyun Ryu, Won Beom Jung, Hackjoon Shim, Sung Min Ko

**Affiliations:** 1 Department of Radiology, Seoul National University College of Medicine, Seoul, Republic of Korea; 2 Medical Imaging AI Research Center, Canon Medical Systems Korea, Seoul, Republic of Korea; 3 Department of Radiology, Wonju Severance Christian Hospital, Yonsei University Wonju College of Medicine, Wonju, Republic of Korea; 4 Korea Brain Research Institute (KBRI), Daegu, Republic of Korea; 5 CONNECT-AI Research Center, Yonsei University College of Medicine, Seoul, Republic of Korea

**Keywords:** Coronary vessel, Multidetector computed tomography, Image reconstruction, Myocardium

## Abstract

**Background:**

The recently introduced super-resolution (SR) deep learning image reconstruction (DLR) is potentially effective in reducing noise level and enhancing the spatial resolution. We aimed to investigate whether SR-DLR has advantages in the overall image quality and intensity homogeneity on coronary computed tomography (CT) angiography with four different approaches: filtered-back projection (FBP), hybrid iterative reconstruction (IR), DLR, and SR-DLR.

**Methods:**

Sixty-three patients (mean age, 61 ± 11 years; range, 18–81 years; 40 men) who had undergone coronary CT angiography between June and October 2022 were retrospectively included. Image noise, signal to noise ratio, and contrast to noise ratio were quantified in both proximal and distal segments of the major coronary arteries. The left ventricle myocardium contrast homogeneity was analyzed. Two independent reviewers scored overall image quality, image noise, image sharpness, and myocardial homogeneity.

**Results:**

Image noise in Hounsfield units (HU) was significantly lower (*P* < 0.001) for the SR-DLR (11.2 ± 2.0 HU) compared to those associated with other image reconstruction methods including FBP (30.5 ± 10.5 HU), hybrid IR (20.0 ± 5.4 HU), and DLR (14.2 ± 2.5 HU) in both proximal and distal segments. SR-DLR significantly improved signal to noise ratio and contrast to noise ratio in both the proximal and distal segments of the major coronary arteries. No significant difference was observed in the myocardial CT attenuation with SR-DLR among different segments of the left ventricle myocardium (*P* = 0.345). Conversely, FBP and hybrid IR resulted in inhomogeneous myocardial CT attenuation (*P* < 0.001). Two reviewers graded subjective image quality with SR-DLR higher than other image reconstruction techniques (*P* < 0.001).

**Conclusions:**

SR-DLR improved image quality, demonstrated clearer delineation of distal segments of coronary arteries, and was seemingly accurate for quantifying CT attenuation in the myocardium.

## Background

Coronary computed tomography (CT) angiography accounts as a first-line noninvasive test to provide a precise evaluation for the diagnosis of coronary artery diseases [1–3]. During the last decade, advances in CT scanners in the form of faster gantry rotation times (250 ms) and a greater number of detector rows (320-detector) have allowed the scanning of the entire heart in a single acquisition [4–7]. In addition, development of deep learning image reconstruction (DLR) has addressed the low signal to noise ratio (SNR) limitations even with a lower radiation dose using deep convolutional neural networks [8, 9]. Unfortunately, due to tradeoffs between image noise and spatial resolution, the accurate interpretability of in-stent lumen, plaque compositions, and severe coronary calcifications are still challenging due to the lower spatial resolution in coronary CT angiography as compared to invasive coronary angiography [10, 11]. Although ultrahigh-resolution CT with a small detector cell size (0.25 × 0.25 mm) allows better visualization of stent, coronary artery calcification, and in-stent stenosis, it has limitations in functions other than spatial resolutions, such as narrower z-axis coverage, longer gantry rotation speed, and lower SNR [12–14]. The super-resolution (SR) DLR (Precise IQ Engine [PIQE], Canon Medical Systems Corp) has been introduced for 320-row CT scanner, which is trained with images acquired using ultrahigh-resolution CT [15]. Previous studies have demonstrated that SR-DLR offers superior image quality with better delineation of cardiac structures and higher sharpness than other image reconstruction methods, including conventional DLR, model-based iterative reconstruction (IR), hybrid IR, and filtered-back projection (FBP) on coronary CT angiography [13, 16–18].

Single first-pass coronary CT angiography enables the detection of hypoattenuation in the myocardial area [19]. Normal myocardial enhancement should be homogeneous to exclude myocardial ischemia [20, 21]. The higher image noise with wider standard deviations generates inhomogeneity across the myocardium, thus hindering the assessment of ischemic cardiomyopathy [22]. As a result, higher image quality is required for the accurate evaluation of myocardial ischemia by decreasing the inhomogeneity of the myocardium. Therefore, SR-DLR would provide better visualize the fine cardiac structure, small vessels, and myocardium.

We investigated the image quality in both proximal and distal segments of coronary arteries and myocardium on coronary CT angiography reconstructed with four different approaches: FBP, hybrid IR, DLR, and SR-DLR.

## Methods

### Patient population

**Fig. 1:**
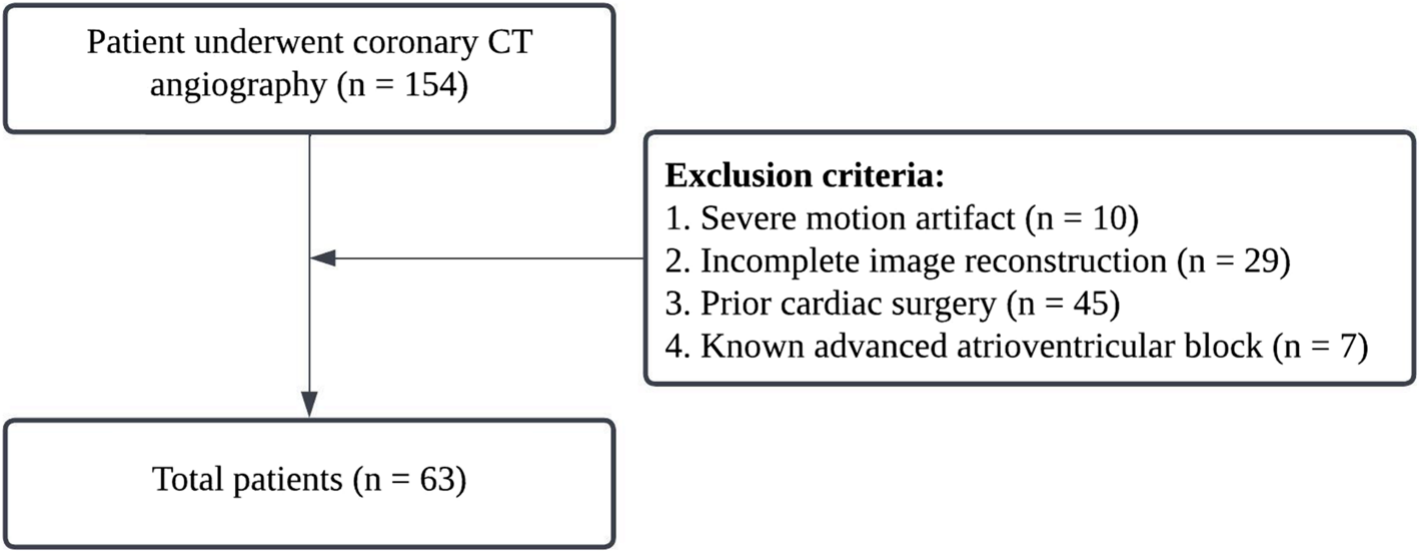
Patient inclusion and exclusion criteria. CT, computed tomography

We assessed 154 patients who had undergone coronary CT angiography from June 2022 to October 2022. Figure 1 demonstrates the study flowchart and exclusion criteria. Patients with severe motion artifacts or artifacts arising from high-attenuation metals or pacemakers (*n* = 10), incomplete image reconstruction due to raw projection data being lost (*n* = 29), prior cardiac surgery or stent placement (*n* = 45), and known advanced atrioventricular block (*n* = 7) were excluded from the study. Thus, evaluation was performed in 63 patients in this study.

### Coronary CT angiography protocol

All patients were examined with a 320-MDCT volume scanner (Aquilion ONE PRISM, Canon Medical Systems Corp) with the following imaging parameters: tube voltage, 100 kVp; gantry rotation time, 275 ms; detector collimation 0.5 mm; thickness 0.5 mm; and field of view, 200 mm. An automatic exposure control with a standard deviation of 20 was used for the tube current. The scan was started with an automatic bolus-tracking program (^SURE^Start, Canon Medical Systems Corp) in the ascending aorta (trigger threshold level, 120 Hounsfield units [HU]). A bolus of 80 mL iopromide contrast material (Ultravist 370, Schering AG) was administered into the antecubital vein at a flow rate of 4.5 mL/sec, followed by 30 mL of saline at a flow rate of 4.5 mL/sec. The contrast medium was injected using a dual-head power injector (Dual Shot Alpha 7, Nemoto Kyorindo Co Ltd). Prospective electrocardiography triggering methods covering 30% to 80% of the cardiac cycle were used for the scanning without additional β-blockers. Image reconstruction methods were performed using FBP, hybrid IR (default kernel, FC43; Adaptive Iterative Dose Reduction 3D, Canon Medical Systems Corp), DLR with the cardiac standard option (Advanced Intelligent Clear IQ Engine, Canon Medical Systems Corp), and SR-DLR with the cardiac standard option (PIQE). Images were processed using the VITREA workstation (Vital Images). The effective radiation dose was calculated by multiplying the dose-length product by the conversion coefficient (0.014 mSv × mGy^−1^ × cm^−1^) [23].

### Objective analysis

The image noise was evaluated as the standard deviation of HU by placing a 100 mm^2^ region of interest (ROI) in the subcutaneous fat of the anterior chest wall. The CT attenuation (HU) of the proximal and distal segments of the major coronary arteries, including the left main coronary artery, left circumflex artery, left anterior descending artery, and right coronary artery, were measured and used to calculate the SNR and contrast to noise ratio (CNR). Each ROIs in the major coronary arteries was as large as possible, while avoiding the inclusion of calcification, plaque, and wall. The CT attenuation (HU) of the pectoralis major muscle on the axial images (ROI, 30 mm^2^) was measured and used to calculate the CNR. SNR and CNR were calculated using the following formulae:

$$\text{SNR}= \frac{{HU}_{vessel}}{{SD}_{vessel}};CNR= \frac{{HU}_{vesssel}-{HU}_{pectoralis muscle} }{Image noise}$$

**Fig. 2:**
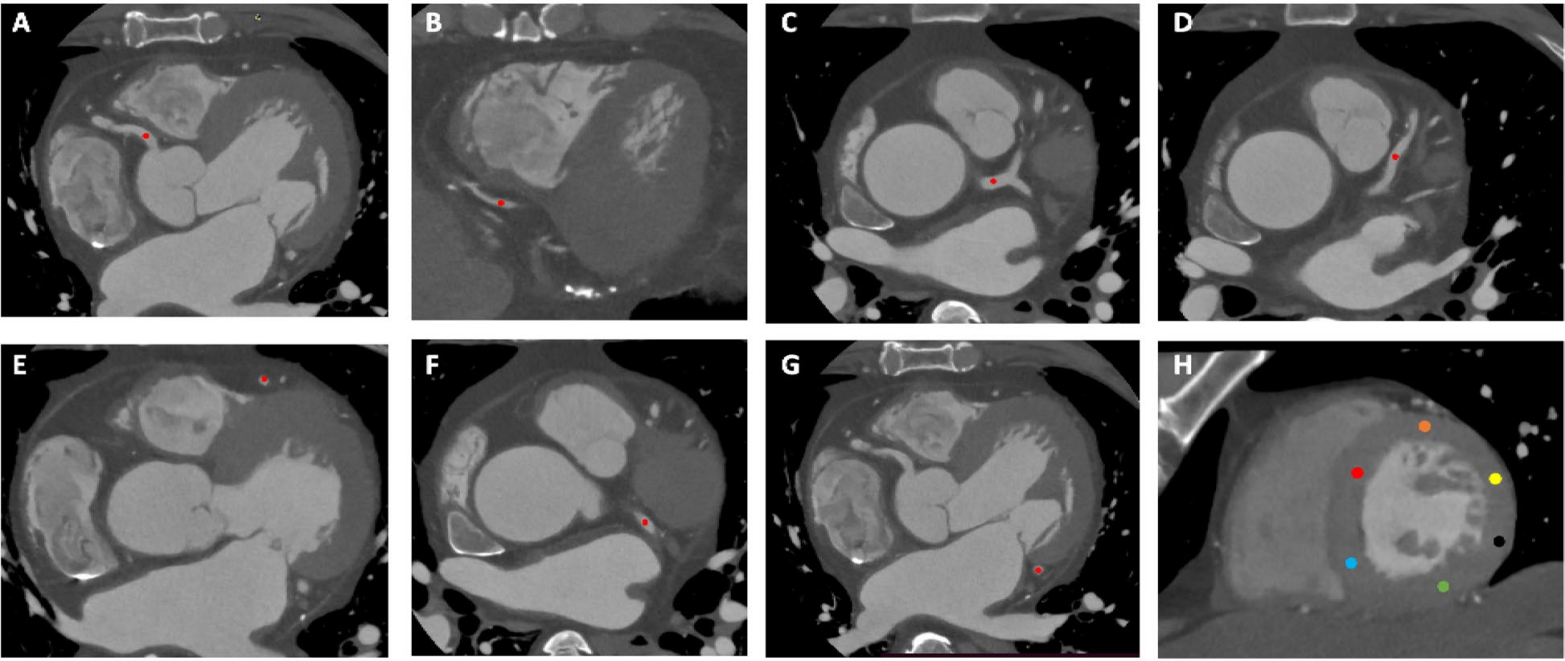
Locations of the regions of interest (ROIs) for signal to noise ratio, contrast to noise ratio, and myocardium homogeneity. **A**–**G** The ROIs in both proximal and distal segments of coronary arteries was as large as possible while avoiding the inclusion of the calcification, plaque, and wall. **H** In addition, myocardial contrast homogeneity was measured by placing the 10-mm^2^ ROI in the inferoseptal (red), anteroseptal (blue), anterior (green), anterolateral (black), inferolateral (yellow), and inferior (orange) of the left ventricle in the midventricular

In addition, short-axis slices of the left ventricle were obtained at the midventricular level, where papillary muscles were seen. Then, left ventricle myocardium contrast homogeneity was analyzed by placing the 10 mm^2^ ROI in the inferoseptal, anteroseptal, anterior, anterolateral, inferolateral, and inferior segments (Fig. 2). Histogram analysis of myocardium CT attenuation was performed using MATLAB ver.8.2.0 (MathWorks) for different image reconstruction methods.

### Subjective analysis

The qualitative analysis was independently evaluated by two experienced readers (a board-certified radiologist with 5 years of experience and a cardiovascular radiologist with 21 years of experience), and both readers were blinded to the four different image reconstruction methods. All images were randomized for subjective assessment. Two readers allowed to adjust windows level and width and randomly performed the image quality in the whole volume data by using a 5-point Likert scale:5 points (excellent overall image quality, minimal image noise, clear visualization of vessel wall definition and high vessel attenuation in both proximal and distal segments of coronary arteries, and minimal myocardium inhomogeneous attenuation), 4 points (good overall image quality, mild image noise, well-preserved vessel wall definition and good vessel attenuation in both proximal and distal segments of coronary arteries, and mild myocardium inhomogeneous attenuation), 3 points (moderate overall image quality, moderate image noise, moderate vessel wall definition and adequate vessel attenuation in both proximal and distal segments of coronary arteries, and moderate myocardium inhomogeneous attenuation), 2 points (poor overall image quality, severe image noise, blurring of the vessel wall definition and low vessel attenuation in both proximal and distal segments of coronary arteries, and severe myocardium inhomogeneous attenuation), and 1 point (very poor overall image quality, very severe image noise, poor vessel wall definition and inadequate vessel attenuation in both proximal and distal segments of coronary arteries, and very severe myocardium inhomogeneous attenuation).

### Statistical analysis

Data normality was tested using the Kolmogorov–Smirnov and Shapiro–Wilk tests. Image noise, SNR, CNR, and myocardial CT attenuation were compared among different image reconstruction methods using one-way analysis of variance, and post hoc Tukey test was performed. Subjective image analysis was compared using the Kruskal–Wallis test for different image reconstruction methods. Interclass correlation coefficient (ICC) with absolute-agreement and two-way random effect was used for the observer agreement, where an ICC value of less than 0.5 denoted poor, 0.5–0.75 denoted moderate, 0.75–0.90 denoted good, and more than 0.9 denoted excellent agreement [24]. *P* < 0.05 was considered statistically significant. All statistical analyses were performed using the IBM SPSS ver. 25.0 (IBM Corp).

## Results

The patient characteristics are shown in Table 1. A total of 63 patients (mean age, 61 ± 11 years; range, 18–81 years; 40 men) were enrolled in this study. The mean body mass index was 25.4 ± 2.8 kg/m^2^. Thirty-four patients (54.0%) had hypertension, while 32 (50.8%) had hyperlipidemia. The mean effective dose was 4.9 ± 0.8 mSv.

**Table 1:** Patient demographic

Characteristics	Value (*n* = 63)
Age (yr)	61 ± 11
Male sex	40 (63.5)
Body mass index (kg/m^2^)	25.4 ± 2.8
Medical history	
Diabetes	9 (14.3)
Hypertension	34 (54.0)
Hyperlipidemia	32 (50.8)
Current and ex-smokers	20 (31.7)
Radiation dose	
CT dose index volume (mGy)	25.6 ± 3.9
Dose-length product (mGy × cm)	355.0 ± 60.2
Effective dose (mSv)	4.9 ± 0.8

Values are presented as mean ± standard deviation or number (%)

*CT* Computed tomography

### Objective analysis

Image noise was significantly lower (*P* < 0.001) for the SR-DLR (11.2 ± 2.0 HU) compared to those associated with other image reconstruction methods including FBP (30.5 ± 10.5 HU), hybrid IR (20.0 ± 5.4 HU), and DLR (14.2 ± 2.5 HU). Post hoc Tukey test showed significant difference in all possible pairwise comparison between different image reconstruction methods.

**Fig. 3:**
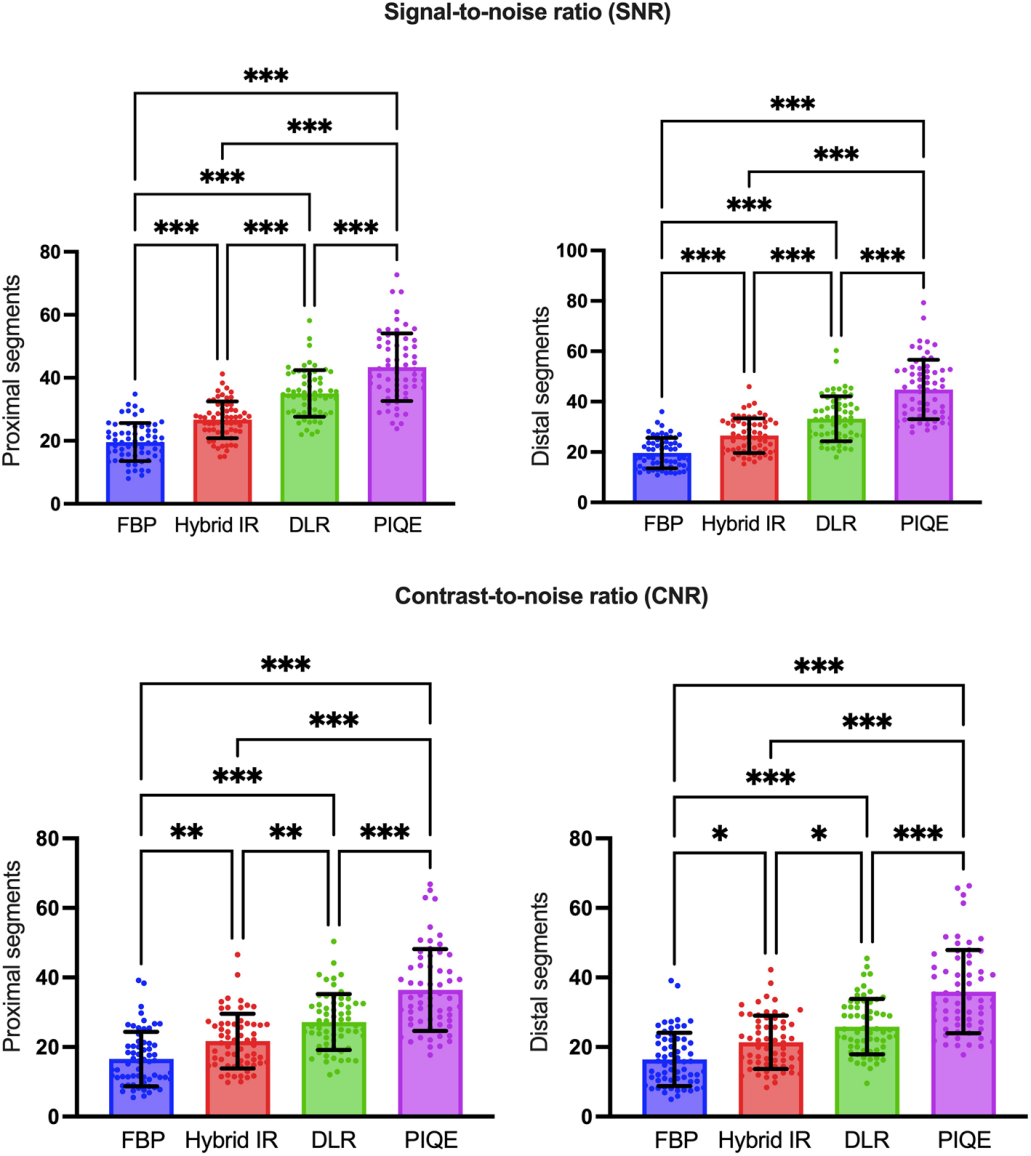
Results of **A**, **B** the signal to noise ratio (SNR) and **C**, **D** contrast to noise ratio (CNR) between different image reconstruction methods. The mean SNR and CNR were significantly higher in both the proximal and distal segments of the coronary arteries for images reconstructed with super-resolution (SR) deep learning image reconstruction (DLR) compared to those associated with other image reconstruction methods, including filtered-back projection (FBP), hybrid iterative reconstruction (IR), and DLR. ^***^P < 0.001, ^**^P < 0.002, ^*^P < 0.033

Table 2 summarizes the results of the SNR and CNR between different image reconstruction methods in the proximal and distal segments of the coronary arteries. In the proximal segments of the coronary arteries, the mean SNR was significantly improved (*P* < 0.001) for SR-DLR (43.4 ± 10.7) than for FBP (19.6 ± 6.0), hybrid IR (26.7 ± 5.8), and DLR (35.0 ± 7.4). In the distal segments of the coronary arteries, the mean SNR was significantly higher (*P* < 0.001) for SR-DLR (44.8 ± 11.8) than for FBP (19.6 ± 6.0), hybrid IR (26.5 ± 6.8), and DLR (33.2 ± 8.9). Furthermore, the mean CNR was significantly higher (*P* < 0.001) for images reconstructed with SR-DLR (proximal segments, 36.4 ± 11.7; distal segments, 35.9 ± 11.9) than for other image reconstruction methods, including FBP (proximal segments, 16.5 ± 7.8; distal segments, 16.4 ± 7.6), hybrid IR (proximal segments, 21.7 ± 7.8; distal segments, 21.3 ± 7.7), and DLR (proximal segments, 27.2 ± 8.0; distal segments, 25.8 ± 7.9). A post hoc Tukey test revealed significant differences between each of the different image reconstruction methods (Fig. 3).

**Table 2:** The results of SNR and CNR between different image reconstructions in the proximal and distal segments of coronary arteries

Parameter	FBP	Hybrid IR	DLR	SR-DLR	*P-*value
SNR					
Proximal					
RCA	20.4 ± 8.0	27.9 ± 10.0	35.0 ± 11.1	43.8 ± 17.6	0.001
LMA	18.2 ± 7.5	25.6 ± 9.0	33.8 ± 10.4	40.8 ± 12.5	0.001
LAD	19.7 ± 7.9	26.4 ± 8.6	35.0 ± 11.2	43.1 ± 13.9	0.001
LCX	19.9 ± 7.7	26.6 ± 8.9	36.1 ± 12.4	45.6 ± 17.9	0.001
Distal					
RCA	17.1 ± 7.4	23.9 ± 9.6	32.1 ± 12.0	42.4 ± 16.1	0.001
LAD	20.7 ± 8.4	27.6 ± 10.4	34.3 ± 13.3	46.4 ± 17.1	0.001
LCX	20.9 ± 8.7	27.9 ± 9.9	33.1 ± 10.8	45.5 ± 19.5	0.001
CNR					
Proximal					
RCA	16.8 ± 8.2	22.4 ± 8.5	27.3 ± 8.7	36.2 ± 12.3	0.001
LMA	16.3 ± 7.6	22.1 ± 7.6	27.6 ± 7.7	36.6 ± 11.4	0.001
LAD	16.6 ± 8.3	22.3 ± 8.3	27.0 ± 8.4	36.5 ± 12.2	0.001
LCX	16.3 ± 7.6	21.6 ± 8.0	26.7 ± 8.3	36.3 ± 12.1	0.001
Distal					
RCA	15.9 ± 7.6	21.2 ± 8.0	25.3 ± 8.3	34.9 ± 12.0	0.001
LAD	17.0 ± 8.1	22.5 ± 8.2	26.4 ± 8.6	36.4 ± 12.5	0.001
LCX	16.3 ± 7.6	21.5 ± 7.8	25.7 ± 7.9	36.3 ± 12.1	0.001

Values are presented as mean ± standard deviation

*SNR* Signal to noise ratio, *CNR* Contrast to noise ratio, *FBP* Filtered-back projection, *IR* Iterative reconstruction, *DLR* Deep learning image reconstruction, *SR* Super-resolution, *RCA* Right coronary artery, *LMA* Left main coronary artery, *LAD* Left anterior descending artery, *LCX* Left circumflex artery

**Fig. 4:**
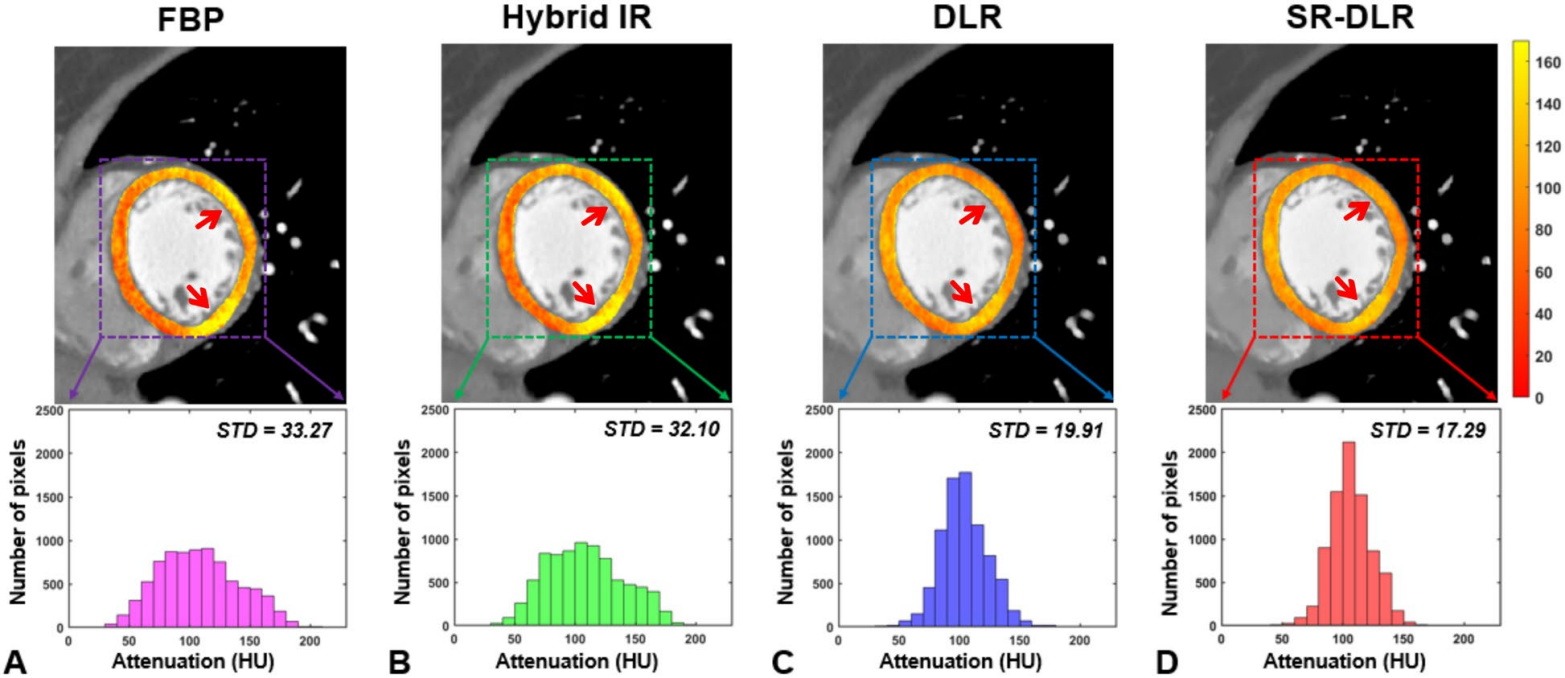
Representative cases with myocardium homogeneity among different image reconstruction methods. The myocardium computed tomography (CT) attenuation resulted in significantly different for filtered-back projection (FBP; *P* < 0.001) and hybrid iterative reconstruction (IR; *P* < 0.001), while there were no significant differences for deep learning image reconstruction (DLR; *P* = 0.153) and super-resolution (SR) DLR (*P* = 0.345). **A**–**D** The color-coded map with left ventricle short-axis images at the midventricular level demonstrated markedly higher myocardium CT attenuation in the inferolateral and anterior segments (arrows) for FBP and hybrid IR while myocardium CT attenuation was homogeneous for DLR and SR-DLR. **E**–**H** Histogram showed wider distribution in attenuation values for FBP and hybrid IR with higher standard deviations (STDs). Conversely, DLR and SR-DLR resulted in narrow histogram distribution in attenuation values with lower STD

The myocardial CT attenuation is shown in Table 3. FBP and hybrid IR resulted in a significant difference in myocardial CT attenuation between different segments of the myocardium (both *P* < 0.001). However, no statistical difference was evident in the myocardial attenuation among the inferoseptal, anteroseptal, anterior, anterolateral, inferolateral, and inferior segments on images with DLR (*P* = 0.153) and SR-DLR (*P* = 0.345). Figure 4 illustrates a representative case of the color-coded map of myocardium CT attenuation among different image reconstruction methods and histogram analysis. The histograms of SR-DLR and DLR exhibit a narrow width, resulting in reduced signal variability and image compared to those of FBP and hybrid IR.

**Table 3:** The results of myocardial computed tomography attenuation among different locations

Location	FBP	Hybrid IR	DLR	SR-DLR
Inferoseptal	92.6 ± 14.9	92.6 ± 14.8	101.2 ± 14.5	102.3 ± 13.0
Anteroseptal	91.2 ± 15.9	91.0 ± 15.8	102.2 ± 14.6	101.9 ± 13.3
Anterior	102.2 ± 17.7	102.4 ± 17.3	97.2 ± 10.6	98.5 ± 12.3
Anterolateral	110.7 ± 17.3	110.3 ± 16.9	99.7 ± 11.1	101.2 ± 11.5
Inferolateral	107.2 ± 22.7	110.1 ± 12.2	94.4 ± 9.4	95.6 ± 9.5
Inferior	110.7 ± 14.7	110.3 ± 14.4	95.9 ± 13.4	99.3 ± 12.6
P-value	0.001	0.001	0.153	0.345

Values are presented as mean ± standard deviation

*FBP* Filtered-back projection, *IR* Iterative reconstruction, *DLR* Deep learning image reconstruction, *SR* Super-resolution

### Subjective analysis

**Fig. 5:**
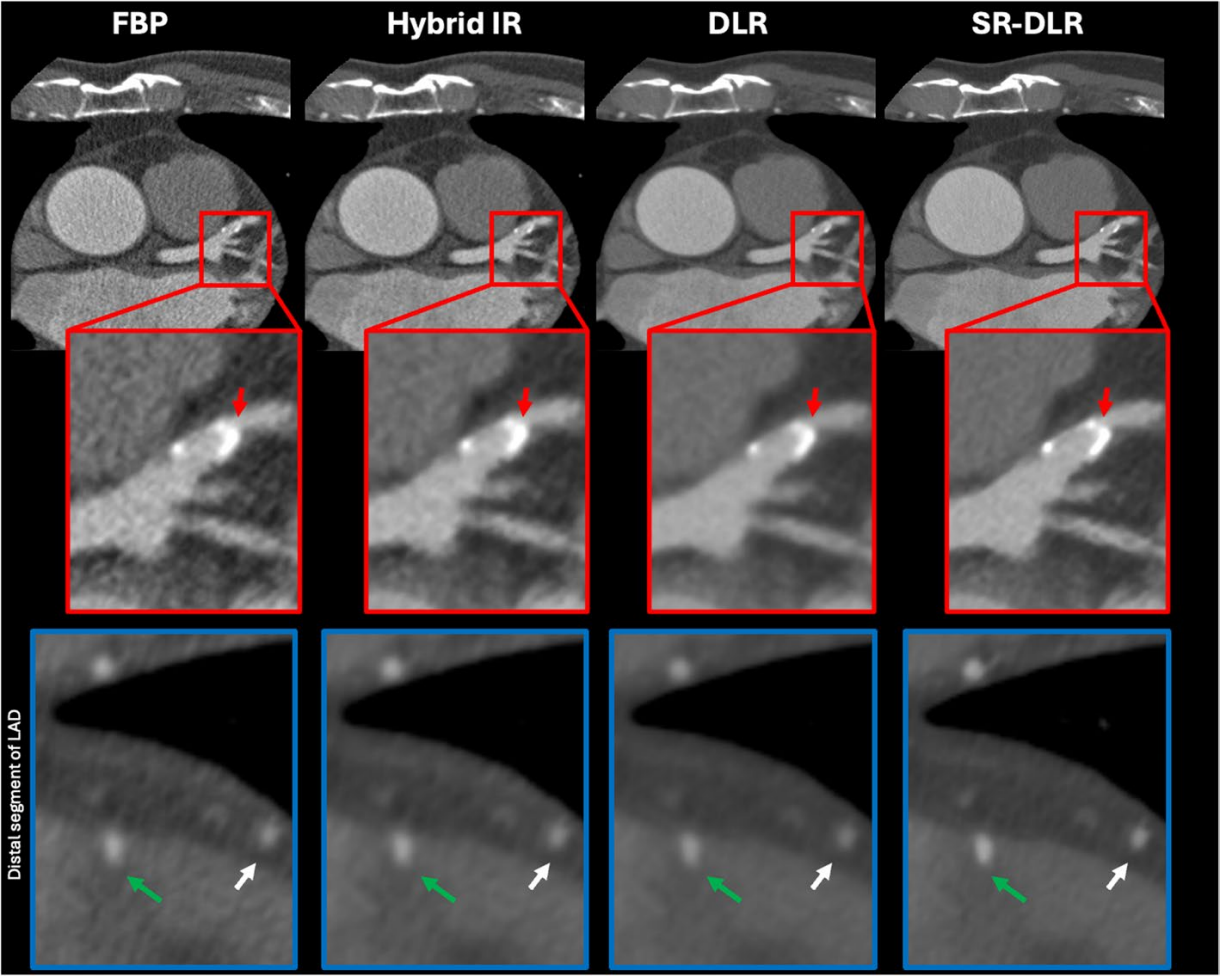
A 63-year-old man with coronary artery calcification and myocardial bridging underwent coronary computed tomography (CT) angiography. The coronary CT angiography images reconstructed using for different methods: filtered-back projection (FBP), hybrid iterative reconstruction (IR), deep learning image reconstruction (DLR), and super-resolution (SR) DLR. Axial images are shown for each reconstruction. SR-DLR demonstrates better delineation of calcification (red arrows) with lower blooming artifacts, sharper branch visualization, and the least image noise compared with other image reconstructions. SR-DLR enhances the delineation of the boundary between the distal segment of the coronary artery (white arrows) and adjacent structure, and it provides better visualization of deep myocardial bridging in the mid left anterior descending artery (LAD; green arrows). Red boxes indicate the magnified image of the proximal segment of the LAD, while blue boxes indicate the magnified image of the distal segment of the LAD

**Fig. 6:**
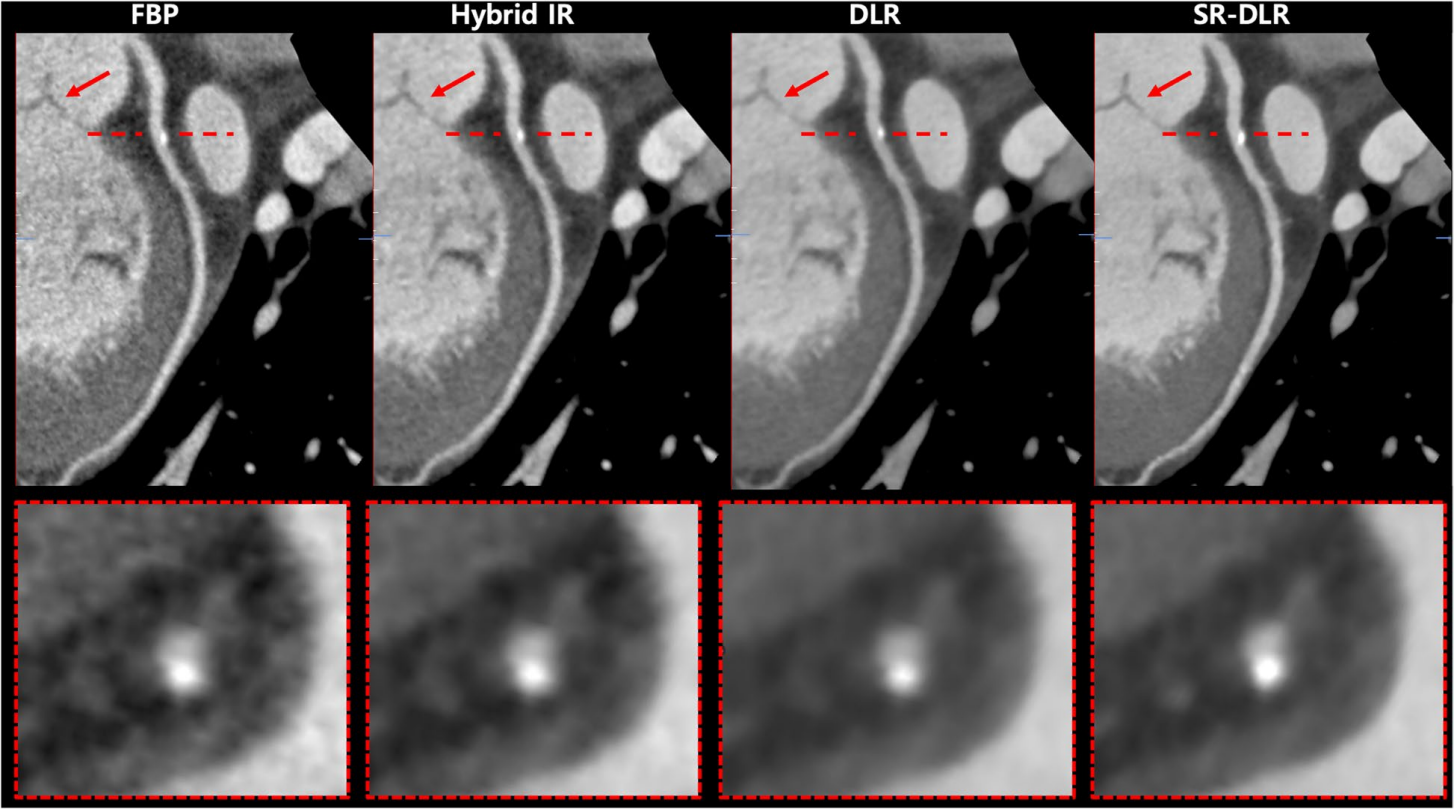
A 72-year-old woman who underwent coronary computed tomography (CT) angiography. The coronary CT angiography images were reconstructed with filtered-back projection (FBP), hybrid iterative reconstruction (IR), deep learning image reconstruction (DLR), and super-resolution (SR) DLR. Curved multiplanar reformatted image are shown for each reconstruction method. SR-DLR provides clearer delineation of the aortic valve (red arrows) and the coronary artery with external calcified plaque (red dashed boxes), while allowing edge smoothness and a noise-free appearance compared to the other image reconstructions

Table 4 summarizes the results of the subjective analysis of the different image reconstruction methods. The overall image quality was scored significantly higher with SR-DLR than with the other image reconstruction methods (*P* < 0.001). In addition, the subjective image analysis for image noise was scored as mild image noise for SR-DLR (4.9 ± 0.2), DLR (4.8 ± 0.3), and hybrid IR (4.3 ± 0.5), while it was scored as moderate for FBP (3.4 ± 0.6; *P* < 0.001). For vessel sharpness, the depiction of both proximal and distal segments and its attenuation were scored significantly higher in SR-DLR images than in FBP, hybrid IR, and DLR images (*P* < 0.001). Lastly, myocardium homogeneity was rated better (*P* < 0.001) for SR-DLR (4.9 ± 0.1) and DLR (4.8 ± 0.3) than for FBP (3.6 ± 0.5) and hybrid IR (4.3 ± 0.5). For interobserver agreement, the ICC was 0.884 (95% confidence interval [CI], 0.849–0.910) for overall image quality, 0.850 (95% CI, 0.805–0.884) for image noise, and 0.799 (95% CI, 0.739–0.845) for image sharpness in the proximal segments. The interobserver agreement between the two readers was excellent (ICC, 0.901; 95% CI, 0.872–0.924) for image sharpness in the distal segments. Representative cases are shown in Figs. 5 and 6.

**Table 4:** The results of subjective analysis among different image reconstructions

Method	Overall image quality	Image noise	Image sharpness		Myocardium homogeneity
			Proximal segment	Distal segment	
FBP	3.5 ± 0.5	3.4 ± 0.6	3.7 ± 0.4	3.4 ± 0.5	3.6 ± 0.5
Hybrid IR	4.2 ± 0.5	4.3 ± 0.5	4.3 ± 0.5	4.1 ± 0.5	4.3 ± 0.5
DLR	4.8 ± 0.2	4.8 ± 0.3	4.8 ± 0.3	4.6 ± 0.5	4.8 ± 0.3
SR-DLR	4.9 ± 0.1	4.9 ± 0.2	4.9 ± 0.1	4.9 ± 0.2	4.9 ± 0.1
P-value	0.001	0.001	0.001	0.001	0.001
ICC (95% CI)	0.884 (0.849–0.910)	0.850 (0.805–0.884)	0.799 (0.739–0.845)	0.901 (0.872–0.924)	0.867 (0.827–0.897)

Values are presented mean ± standard deviation of the two observers

*FBP* Filtered-back projection, *IR* Iterative reconstruction, *DLR* Deep learning image reconstruction, *SR* Super-resolution, *ICC* Interclass correlation coefficient, *CI* Confidence interval

## Discussion

The results of this study demonstrate that SR-DLR achieves higher image quality, better visualization of small distal segments of coronary arteries, and minimum inhomogeneous myocardium attenuation on coronary CT angiography than those associated with FBP, hybrid IR, and DLR. To our knowledge, the present study is the first to analyze the image quality and visualization of distal coronary vessels and myocardial inhomogeneity on coronary CT angiography with SR-DLR.

Multiple studies have investigated the effects of DLR across various sections compared to those of FBP and hybrid IR in clinical and phantom studies [25–28], reporting that the DLR yields superior reduction of image noise, improvement in the SNR, CNR, and fewer artifacts with higher diagnostic accuracy against FBP and hybrid IR. Interestingly, the present study showed that the mean SNR and CNR in the coronary arteries improved by 29.2% and 36.4% with SR-DLR, respectively, compared to those with DLR. Both observers also favored SR-DLR in terms of overall image quality and image noise compared to those associated with other image reconstruction methods. The image noise was reduced by approximately 21.4% with SR-DLR compared to other image reconstructions which was consistent with previous studies [13, 17, 18, 29]. The detectability of plaques with less than 50% stenosis and delineation of the stent strut was superior in SR-DLR compared with other image reconstructions in previous studies [17, 29]. On the other hand, the present study investigated the proximal and distal segments of coronary arteries and myocardium homogeneity. Noticeably, SR-DLR showed a lower image noise in both proximal and distal segments of coronary arteries as well as higher SNR, CNR, and qualitative analysis assessment in present study. According to the European Society of Cardiology, complete revascularization is not required for small vessels (< 2 mm) [30]. The high risk of restenosis after percutaneous coronary intervention in small vessels indicates the importance of accurately evaluating the vessel size [31, 32]. However, by introducing newer generation drug-eluting stents for percutaneous coronary intervention in small vessels, SR-DLR may improve the visualization of small vessels [33, 34]. Theoretically, spatial resolution affects image noise; as the spatial resolution increases, image noise increases [12]. In this study, the difference between DLR and SR-DLR is the training process for deep convolutional neural networks. A three-dimensional (3D) deep convolutional neural network was used for the training process of SR-DLR, whereas 2D was utilized for DLR. Thus, using 3D deep convolutional neural networks and downsampling ultrahigh-resolution CT images decreased the image noise and increased the spatial resolution for SR-DLR compared to those of DLR. Moreover, the visualization of coronary artery vessels and side branches was significantly improved with SR-DLR compared to other image reconstruction methods, including FBP, hybrid IR, and DLR. This suggest that SR-DLR can assist in accurate preprocedural percutaneous intervention treatment planning and save procedural time.

Over the last decade, dynamic myocardial CT perfusion was introduced, which has allowed the evaluation of myocardial ischemia [20]. The injected iodinated contrast agent directly perfuses the myocardium. Higher image noise can result in inhomogeneous attenuation of the myocardium and may misdiagnose myocardial ischemic disease when performing dynamic myocardial CT perfusion. When compared with FBP and IR, both SR-DLR and DLR was learned to reduce the image noise using target image at high dose and high image quality and trained to distinguish between the signal from the noise. Thus, the myocardial attenuation was consistent among different locations with DLR and SR-DLR, whereas significant differences were observed in both FBP and hybrid IR. Figure 3 demonstrates a narrower histogram distribution of attenuation values with lower standard deviations for DLR and SR-DLR.

Microcalcification and low attenuation noncalcified plaques are considered powerful predictors of plaque rupture [35]. Our representative cases indicate that the presence of both calcified and noncalcified plaque lesions was better visualized by SR-DLR compared to those visualized by other image reconstruction methods. Higher spatial resolution decreases the blooming artifacts from microcalcification, which would be a strength of SR-DLR for the precise visualization and quantification of plaques. We also believe that the application of SR-DLR can help better identify high-risk plaques in the coronary artery and allow the identification of patients at high risk for cardiovascular diseases. The homogeneous CT attenuation with lower standard deviations among different segments of the myocardium of DLR and SR-DLR could be accurate imaging reconstruction methods for dynamic myocardial CT perfusion for further investigation of myocardial pathology including symptomatic myocardial ischemia without coronary artery obstructive changes, myocardial fibrosis, and microvascular dysfunction.

Our study had several limitations. This was a retrospective, single-center study. All results were limited to one vendor. In addition, we did not evaluate the impact of lower tube voltage or different strengths and options of image reconstruction on image quality. In this study, myocardial inhomogeneity was only evaluated at the midventricular level, where all three major coronary arteries are supplied. Future studies should include more patients with and without myocardial ischemia. Finally, this study focused on the visualization of the proximal and distal segments of normal coronary arteries and normal myocardium homogeneity, and future studies should investigate whether SR-DLR can increase the diagnostic accuracy of coronary artery stenosis by including more patients who underwent invasive coronary angiography.

## Conclusions

SR-DLR improved image quality, demonstrated clearer delineation of distal segments of coronary arteries, and appeared to be an accurate imaging reconstruction method for the quantification of myocardial CT attenuation.

## Data Availability

Not applicable.

## Abbreviations

CI: Confidence interval
CNR: Contrast to noise ratio
CT: Computed tomography
D: Dimensional
DLR: Deep learning image reconstruction
FBP: Filtered-back projection
HU: Hounsfield units
ICC: Interclass correlation coefficient
IR: Iterative reconstruction
PIQE: Precise IQ Engine
ROI: Region of interest
SNR: Signal to noise ratio
SR: Super-resolution

## Data availability

No datasets were generated or analysed during the current study.
